# Drug Delivery Through Placenta and Then Breastmilk for Fetal Cystic Fibrosis: Collateral Benefit and Social Good Do Not Make an Acceptable Risk: Benefit Ratio

**DOI:** 10.1080/15265161.2025.2543720

**Published:** 2025-09-18

**Authors:** Teri L. Hernandez, Michael V. Zaretsky, Brian M. Jackson, Gianna G. Morales, Curtis R. Coughlin, David Badesch, Matthew DeCamp

**Affiliations:** a University of Colorado Anschutz Medical Campus

## CASE INTRODUCTION

The proposed clinical trial is an investigator-initiated non-randomized, two-arm comparative prospective study (Dawson et al. 2025). Careful ethical consideration of risks and benefits is necessary. Here we focus on two questions: (1) Do collateral benefits “count” in the risk-benefit assessment for the pregnant person and after delivery?; and (2) To what extent, if any, should the social context influence ethical decision making? In our assessment, despite the potential for collateral benefits to the healthy volunteer, limited generalizability of benefits to the fetus/infant that would contribute to social good, and concerns over voluntary and fair inclusion make the study as proposed unacceptable—especially in the period after birth.

## ALTRUISM AND COLLATERAL BENEFITS

In this trial, the person who is pregnant and then provides the drug via breastmilk is a healthy volunteer. We ought not assume that this individual experiences all risk and no benefit. True, the side effects of ETI are significant enough that many living with CF are unable to tolerate the therapy (Bathgate et al. 2023). However, pregnant people and new parents are highly motivated by altruism to positively influence the health of their unborn child and newborn. This altruism requires addressing the role of “collateral” benefits of participation, which are not included in conventional risk-benefit assessments (van Rijssel et al. 2025). Collateral benefits have been defined as, “individual benefits arising from study participation, but not from the intervention that is studied,” (p.4) (van Rijssel et al. 2025) including individual knowledge gains (i.e., related to the disease), and psychological benefits (i.e., hope and altruism).

In this circumstance, the person who is pregnant and then provides the drug via breastmilk may benefit (collaterally) by helping to understand the disease affecting their child and others’, or from improved psychological health through the study. Indeed, because the fetus has the potential to benefit from ETI treatment through the pregnant person–and the fetus while *in utero* is part of the pregnant person—psychological benefit might not be a collateral benefit at all; it might be direct. Expectant parents experience stress and anxiety if their fetus requires *in utero* therapy (Arabin et al. 2021), just as parents of critically ill children can experience moral distress (Mooney-Doyle and Ulrich 2020). Thus, the potential maternal psychological benefits of participation, either collateral or direct, may count favorably in the risk: benefit ratio.

## THE ROLE OF SOCIAL CONTEXT

Of course, recognizing collateral benefits is important but not the whole story. For clinical research to be ethical, the risks imposed on participants must also be balanced by the potential for benefits to science and/or society (van Rijssel et al. 2025)—i.e., to social good (Rid and Wendler 2011; Wendler and Rid 2017). Ensuring that research studies are rigorous, methodologically sound, and hold the prospect for generalizability are essential to risk assessment (Rid and Wendler 2011). These standards should not necessarily be different for pregnant people and fetuses/newborns; their inclusion in research is a critical social need (Ren, Bremer, and Pawlyk 2021). The pregnant person, fetus, and newborn remain “therapeutic orphans” because they are continually excluded from research due to perceived risks (Davis and Norwitz 2019).

What about benefits to the fetus and then newborn and generalizability of these benefits? With ETI treatment, there is evidence of direct benefit from ETI therapy to the fetus and then newborn with CF (Bonnel et al. 2025). Although there is a potential for direct benefit both in utero and after delivery, a case can be made that benefits are not maximized, particularly for the newborn. Transplacental ETI therapy even with a defined dose administered to the pregnant person - can result in variable drug concentrations reaching the fetus (due to variability in placental tissue barriers, maturation, and membrane integrity (Sharma et al. 2021)), but few alternative modes of administration exist. Translactation ETI is another matter. Instead of accepting variable transmission via breastfeeding, a fixed dose of the drug could be administered in formula or expressed breast milk. Thus, even though the proposed trial has a prima facie positive risk: benefit ratio for the fetus (as well as the pregnant participant described above), failure to maximize benefits in the form of generalizable doses, particularly after birth, calls the current study design into question. Why not directly administer standardized doses of ETI to the newborn instead of through breastfeeding?

Some might say the reason *not* to directly administer ETI to the newborn relates to the social context of this research, and that our risk: benefit assessment rushed to judgment. After all, cystic fibrosis remains a life shortening disease, and radically improving the life course for a fetus/newborn with CF is highly significant. However, ETI is not approved for children <2 years of age and is expensive. Outside of research, pregnant people may choose to take ETI off-label at the adult dose to help their unborn fetus with CF, even though they risk experiencing potential harm and treatment side effects, have no guarantee of treatment efficacy, and often pay high out-of-pocket costs. Given this context, why not relax our risk: benefit assessment of the newborn period, perhaps for the sake of providing access to ETI and taking advantage of an intriguing natural experiment to collect data about efficacy (or inefficacy) more systematically than an observational study of this real-world practice?

We acknowledge that social context drives research; for example, current standard-of-care therapies, the desire for higher value interventions, costs, and more, shape research questions and design. However, there are several problems with this line of thought. The case does not state who will pay for ETI, but if we assume that burden falls on the pregnant person’s insurance or pocketbook, an issue of fairness arises because only those who can afford to pay can enroll in the treatment arm. On the other hand, if the ETI is provided, concerns over both undue inducement and fairness arise because some healthy people, during pregnancy and after birth, might agree to participate solely because they could not afford the ETI outside of research.

Taken together, allowing the social context of high-cost medications, lack of access, and lack of will to conduct a direct dosing study through formula or expressed breastmilk in the newborn period fails to maximize generalizable dose benefits. Allowing this degree of social context influence over research could inadvertently result in a backdoor approach to permitting all sorts of risky studies, not just during pregnancy.

## CONCLUSION

Although the ethical analysis of this study initially suggests a favorable risk: benefit ratio (especially once collateral benefits associated with maternal altruism and psychological health are included), more careful analysis reveals that the benefits are not maximized or generalizable, especially in the newborn portion of the study. Instead of translactation administration, direct drug dosing of the infant should be considered as a route of drug delivery most likely to benefit the infant and science, with generalizable benefits to the larger population. Although we are sympathetic to concerns about the high cost of medications, lack of access, and the “real-world” practice of translactation ETI delivery, they do not weigh so heavily as to sway our risk: benefit assessment as favorable.
